# The 5 K run in popular fiction: Reading about parkrun and couch to 5 K

**DOI:** 10.3389/fspor.2023.1031934

**Published:** 2023-02-13

**Authors:** Ellen Turner

**Affiliations:** Lund University, Lund, Sweden

**Keywords:** running, fiction (narrative), couch to 5K, parkrun, health promotion

## Abstract

Recent years have witnessed great interest in mass-participation running events (1), and organisations such as parkrun and fitness programmes like Couch to 5 K, have been instrumental in enabling participation for inexperienced runners. Concomitant with this has been a number of fictional works which centre on the 5 K run. I contend that exploring fictional texts can offer a fresh take on how movements such as parkrun and Couch to 5 K have entered the popular imagination. The four texts explored are Wake's *Saturday Morning Park Run* (2020), Park's *A Run in the Park* (2019), Boleyn's *Coming Home to Cariad Cove* (2022), and James's *I Follow You* (2020). The analysis is arranged thematically around the categories of health promotion, individual transformation, and community building. I argue that these texts frequently operate as health promotion tools and can help familiarise would-be runners with how parkrun and Couch to 5 K work.

## Introduction

In the years preceding the COVID-19 pandemic, recreational mass-participation running events enjoyed a huge and dedicated following (1). The 5 K distance, in particular, attracted many devoted runners; exemplifying this, participation in parkrun, the free weekly 5 K event held at various locations both in the UK and internationally, peaked shortly before the March 2020 lockdown with parkrun UK reporting that on Saturday 11 January, 650 events took place in the UK with a total of 193, 746 athletes and 15,357 volunteers taking part (2). The pandemic temporarily halted this Saturday tradition, and though participant numbers are yet to return to their pre-pandemic levels, parkrun UK statistics show that typically over 700 events in the UK take place weekly, with somewhere in the region of 120,000 athletes and 15,000 volunteers regularly in attendance (2). When COVID-19 restrictions meant parkrun and other group fitness activities were suspended, the demand for do-it-yourself fitness tools not surprisingly increased. In July 2020, the NHS reported that from March to June of the same year, over a million people had downloaded the Couch to 5 K fitness app produced by Public Health England (3). This figure represented an over 92% increase on downloads of the beginner-running programme during the same period of the previous year. Simultaneous with this has been an explosion of scholarly interest in parkrun and its role at both an individual and social level (4, 5) and the Couch to 5 K movement is also fast gaining ground in terms of eliciting scholarly attention (6, 7).

Interest in both Couch to 5 K has been evidenced by widespread press coverage and a number of non-fiction publications on the topic,^1^ as well as a significant body of (mostly genre) fiction which too draws on the 5 K movement, often explicitly making reference to parkrun or Couch to 5 K. In addition to the four novels analysed in this article, these works of fiction include Spellman's *Running into Trouble* (2020), Clegg's *The Summer Holiday* (2021), Greenwood's *The Soul Killer* (2020), Enfield's *After Beth* (2021), There are however, to the best of my knowledge, no extant studies which explore these movements from a literary perspective. It is my contention that exploring fictional texts can offer a fresh take on how movements such as parkrun and Couch to 5 K have entered the popular imagination and potentially also pave the way to beginning to explore how readers participate in such narratives. Billington has noted that the past two decades have witnessed unprecedented scholarly interest in the connection between the reading of fiction and wellbeing [(8) p. 1], and this present project aims to contribute to this body of research. Underpinning this is the idea that understanding how ideologies and belief systems within certain communities of practice related to health and fitness might be constructed, reflected, reinforced, and challenged within popular texts can be significant in understanding how individuals and society produce, reproduce, and engage with discourses of mental and physical wellness. As such, this project is inherently entangled with contemporary debates surrounding the individual vs. the collective (9), in its move to reframe the focus of scholarship on public health “less on health behaviour and more on social practice” [(10) p. 239]. By engaging in a shared process of learning within a defined domain, running communities can be viewed as “communities of practice” with a “shared repertoire of resources: experiences, stories, tools, ways of addressing recurring problems” (11). This article considers reading about Couch to 5 K and parkrun in popular fiction as one element in the creation of the “collective tacit knowledge” [(10) p. 250], the unspoken set of rules, which guides thinking and behaviour in specific running communities.

In this article, I am interested in how the 5 K run, in particular, parkrun and the Couch to 5 K initiative, has been represented in recent fictional offerings, with the aim of providing some insight into how the ideologies associated with the 5 K running initiatives are both challenged and reinforced in these texts. The four texts around which much of the discussion here is based are Jules Wake's *Saturday Morning Park Run* (2020), David Park's *A Run in the Park* (2019), Darcie Boleyn's *Coming Home to Cariad Cove* (2022), and Peter James's *I Follow You* (2020). Wake's feel-good novel *The Saturday Morning Park Run* positions parkrun as a panacea to the problems of modern life, and Park's novella (2019) *A Run in the Park,* similarly charts the redemptive power of the couch to 5 K journey. Likewise, for Boleyn Couch to 5 K is transformative for several of the main characters in the novel who are experiencing major upheavals in life. For Wake, Park, and Boleyn, the 5 K journey is a unifying force both thematically and formally; the novels are *about* the 5 K run, with the narrative arc plotted along the 5 K journey. Peter James's crime novel serves as a counterpoint to the positive portrayal of the 5 K run in the above three texts and instead draws on the potential dark side of social media and fitness apps, painting a much murkier picture of parkrun which, rather than being a safe and inclusive space, becomes menacing.

This paper first gives a brief overview of parkrun and Couch to 5 K, along with a succinct survey of relevant scholarly literature. The ensuing analysis, which takes the form of close readings of the four selected novels using tools from literary analysis, is arranged thematically based on the key areas of interest that emerged during a review of current scholarship in sports science and public health which focuses on parkrun and Couch to 5 K. The themes that I base my analysis on are health promotion, individual transformation, and community building. What emerges from the analysis is that the novels explored here tend to take a didactic tone, setting out for the reader the inner workings of the running movements. This leads to fictional texts which resemble quasi-instructional manuals which might have the function of divulging collective tacit knowledge to an implied running community.

## The 5 K movement

The 5 K run is the shortest common long-distance road race distance and thus represents a more manageable goal than, for instance, a half or full marathon, even for those who have previously been adverse to running. Indeed, the couch to 5 K programme was developed with complete beginners in mind, and parkrun's motto is that “It's for everyone” regardless of “whether you walk, jog, run or volunteer.” The decision to explore parkrun and Couch to 5 K in this present paper was in part motivated by the fact that these were the two most frequently named movements in recent fictional work with a central 5 k run trope. But further to this, recent research suggests that these are the two most widely recognised tools for helping people into the world of running. In a 2021 survey exploring general practitioners' awareness of physical activity promotion, it was found that “‘Couch to 5 K’ and the ‘parkrun practice’ initiative were the most widely known and used” physical activity promotion tools (12). When combined, parkrun and Couch to 5 K have a significant market share when it comes to beginner running initiatives and the impact and influence that they have had is reflected in their representation in popular fiction.

The NHS market their Couch to 5 K programme as “a running plan for absolute beginners” stating that “[i]t was developed by a new runner, Josh Clark, who wanted to help his 50-something mum get off the couch and start running, too” (13). The plan is aimed at gradually building fitness — in line with WHO's 2020 guidelines for adults to gradually build their physical activity beginning with small amounts — and is marketed for “everyone”: “Whether you've never run before or you just want to get more active, Couch to 5 K is a free and easy way of getting fitter and healthier” (13). The app provides participants with a virtual coach and was designed to help new runners track their progress to the 5 K target over a 9-week period. Although it was the NHS app that was popularised in the pandemic era, there are numerous other couch to 5 K apps on the market, and the basic 9 week programme is frequently utilised by personal trainers and in-person running groups. Couch to 5 K then is both a programme that individuals can partake in independently, but it can also facilitate group exercise.

Parkrun is a weekly free timed 5 k run that takes place on Saturdays at various locations around the globe. From its humble beginnings as *Bushy Park Time Trial* in London in 2004, where 13 runners embarked on what is now seen as the inaugural parkrun, the movement has exploded (14). Even though parkrun attracts world-class athletes, the parkrun philosophy is that it is for everyone, walkers, joggers, and runners alike. Parkrun has, since 2018, been part of a social prescribing initiative. The parkrun practice initiative was launched by the Royal College of General Practitioners. GPs were therefore able to prescribe parkrun UK to those patients who would be likely to benefit from physical activity (15). The goal of parkrun, however, goes far beyond the physical, with Hindley in his monograph on the event, suggesting that its name with *run* at its heart is “misleading” quoting Nick Pearson, the Global Chief Executive Officer of parkrun as designating parkrun instead as “a social intervention masquerading as a running event” (16).

## Literature review

Research has consistently shown that there is a positive correlation between physical activity and disease prevention” (Fletcher, Landolfo, Niebauer, Ozemek, Arena, & Lavie*,* 2018, p.1622) (17). p. 1622] Consistent with this, the World Health Organization's 2020 guidelines recommend that everyone should exercise, advising that “[a]dults should do at least 150–300 min of moderate-intensity aerobic physical activity; or at least 75–150 min of vigorous-intensity aerobic physical activity; or an equivalent combination of moderate- and vigorous-intensity activity throughout the week, for substantial health benefits” (18). One feature that both parkrun and Couch to 5 K share, is that they are both invested in promoting the health and well-being benefits of exercise in accordance with WHO's 1986 Ottawa Charter in which health promotion is defined as “the process of enabling people to increase control over, and to improve, their health.” Such a focus on personal responsibility has dominated public health discourse from the middle of the twentieth century to the present day with a “renewed emphasis on lifestyle change” [(19) p. 269]. Frequently, health promotion strategies have rested on “responsibilising” approaches. According to Brown, Maslen, & Savulescu though, such an approach places undue moral responsibility on individuals for their state of health and can sometimes imply moral failings for those whose health suffers as a result of their individual behaviours [(20) p. 114]. However, though there might be arguments in favour of such an approach, scholarship has increasingly found health responsibilisation problematic and unjust particularly in its “reframing of social problems as individual ones” [(19) p. 270].

Nevertheless, scholarly research on both Couch to 5 K and parkrun frequently refers to the individual health benefits reaped through engaging in the kind of regular activity encouraged by both. For instance, McKendrick, Bowness, and Tulle remark that parkrun targets “previously less active population groups to address contemporary public health problems such as obesity and cardiovascular disease” and claim that motivations for participating in the event are often connected to a desire to manage one's body in terms of health [(21) p. 2;17]. The potential of parkrun to have a positive impact on the health of both existing athletes for whom it might serve as motivation for continued good habits has been remarked upon (22) but more frequently mentioned is its “untapped potential as a setting for [physical activity] promotion and for public health gain” in groups traditionally less likely to participate in regular exercise “such as women, older adults or those with overweight or obesity, who are at higher risk of being inactive and having chronic disease” [(23) p. 14].

There is less by way of research on the Couch to 5 K programme, but what does exist points to similar health benefits (6). In a 2022 doctoral thesis, Thomson explains that for women participating in jogscotland couch to 5 K groups “achieving physical health” is a key motivating factor for beginning the program (24). Although the goal of improving physical health is central to participants of both parkrun and couch to 5 K, wellbeing goes far beyond just this dimension. For instance, the mental health benefits of parkrun too are frequently cited (25, 26), with much of this value seemingly derived from the supportive community that parkrun provides. Juxtaposed to the substantial body of research which explores the individual in relation to 5 K movements, is that which seeks to examine the embodied experiences of participants so as to, in the words of Wiltshire, Fullagar and Stevinson (2017), “reposition individuals within a collective context” [(27) p. 14]. Consistent with such an approach, Nettleton and Green argue that in order to understand engagement in physical activity, we should look to the construction of “collective tacit knowledge” [(10) p. 250]. To gain insight into the complex set of forces that motivate individual participation in physical activity, it is important to read beyond codified explicit knowledge, in this case, the kinds of easily communicable knowledge available in official promotional material and guides. Implicit and less tangible knowledge tends to be transmitted through social networks and is also worthy of investigation. Couch to 5 K participants and parkrunners, respectively, form “communities of practice” in that they are comprised of “groups of people who share a concern or a passion for something they do and learn how to do it better as they interact regularly” (11).

One study shows that participants of parkrun share a feeling of being “in it together” in relation to both the “physical act of running, and a shared sense of accomplishment” [(25) p. 118]. This finding is supported by Wiltshire, Fullagar, & Stevinson (2017) who argue that the “in it together” mentality can work “to ameliorate the individualising effects of health” [(27) p. 14]. This sense of community is also something fostered in the couch to 5 K programme. Thompson writes that important in couch to 5 k running group participation are considerations around safety and the supportive and non-judgemental nature of groups (24). Furthermore, this sense of community is not only a feature of couch to 5 K running groups, but can also apply to runners using the app independently. In an opinion piece for Manchester Metropolitan University, sports psychologist Dr Wood attributes the popularity of the Couch to 5 K app during lockdown to the fact that social connections are key motivators to participation in physical activity. Wood goes on to write that the app “includes thousands of others on the same journey, and whilst during lockdown many are literally running on their own, they still feel a part of a much larger community of runners which keeps that sense of connectedness” (28). In much the same way that Kurtoğlu-Hooton (2021) has recognised online networks form the backbone for ultrarunning “communities of practice” (29), online groups have helped foster 5 K communities also. This is supported by findings from Tallarita which suggest that parkrun's positive impact was felt throughout the pandemic because of the digital networking tools that enabled the community to still connect even when physical events were cancelled (30). From a social practice perspective, investigating the ways in which such networks participate in “collective tacit knowledge” construction (31) is crucial to understanding what makes “certain practices more or less likely” [(10) p. 250] and thus central to initiating change.

In addition to community building, other key themes that have emerged in scholarship on parkrun, in particular, are that of accessibility and inclusivity. As Hindley writes, participation is a central part of the parkrun ethos”, with the success of the organisation being in part attributable to “its simplicity and accessibility” [(1) p. 87]. Similar sentiments are echoed by Reece, Quirk, Wellington, Haake, & Wilson who discuss the fact that participation is promoted particularly amongst traditionally less active sectors of society with events being launched, for instance, in prisons and disadvantaged areas and efforts being made to ensure events are accessible for those with disabilities [(32) p. 327]. Social prescribing initiatives in which general practitioners actively promote couch to 5 K groups and parkrun are also designed to target those who might previously have been non-runners. It is this individual transformation from non-runner to runner, from inactive to active, from unhealthy to healthy that is the final theme that I will explore in this literature review, and it is one that, as the latter analysis here will demonstrate, is central in the fictional texts that I explore in this article. As McKendrick, Bowness, and Tulle affirm, “parkrun appeals to non-runners, who, after engagement with the practice, become a ‘runner’” with the “public health potential of parkrun” resting in “its ability to catalyse these ‘identity shifts’” [(21) p. 21] Moreover, couch to 5 K and parkrun not only facilitate an identity shift from non-runner to runner, but also from solitary individual exerciser, to member of a running community (33).

## Theoretical background

The therapeutic possibilities of literature and the notion that reading fiction might promote well-being are the topic of much scholarship. In the 1980s Northrop Frye supposed that the inseparable nature of mind and body meant that “imaginative constructs” like literature could “have a direct role to play in physical health” [(34) p. 469]. Although as Bate and Schuman argue in their *Lancet* article, measuring the impact of reading on health and wellbeing is notoriously difficult because of the very personal nature of reading (35), other research suggests that it is the reflection and discussion on reading, rather than the reading itself, that lends itself to wellbeing gains (36). Shared reading groups, in particular, have been proposed as a particularly effective way to harness literature's therapeutic value (8). It is beyond the scope of this article to delve into whether reading literature can impact the wellbeing of readers directly, but previous research hints at the possibilities of collective reading practices as a useful mechanism for wellbeing enhancement. For this project, it is particularly interesting that the emphasis here is on community building, since this is also one of the key goals of the 5 K movements discussed here also.

Where popular novels about running fit into the conversation about engagement in physical activity is that they not only offer possibilities for reflection on core themes as outlined in the previous section, but they are also part and parcel of the creation of “collective tacit knowledge” (31). Underpinning this current paper is the assumption that narrative, as Bruner articulates, informs how human experience is comprehended [(37) p. 21]. Narrative conventions are complex since they are “transmitted culturally” and represent a particular “version of reality whose acceptability is governed by convention and ‘narrative necessity’” [(37) p. 4]. Narratives are never “ethically neutral” [(38) p. 115], and the stories that we read, as well as the stories that we tell, are informed by various socially transmitted sets of values (39). Popular novels can function a medium for the diffusion of tacit running community knowledge, reinforcing the collective values and cementing identities of existing 5 K runners, and acculturating “outsiders” to such ideologies. The first step to exploring the untapped potential in unravelling the relationship between consuming fiction, and the generation on collective tacit knowledge is to examine the messages conveyed in the novels themselves. Although this project does not extend to investigating reader engagement, part of its rationale rests on the promise that such engagement could offer.

## Methodological considerations

In achieving the goal of unravelling the messages conveyed in popular fiction, this paper makes use of tools from literary studies to analyse aspects such as characterisation, narrative perspective, and use of figurative language to perform close textual readings of the four selected novels. In doing so, this paper builds on interdisciplinary methodological traditions which combine tools from literary scholarship to perform sociologically informed readings. Bairner argued for the case that sports fiction can to a certain extent “meet the challenge of uncovering sociologically valuable insights” [(40) p. 524]. Furthermore, Bairner & May assert that novels represent “ideal vehicles for examining societies, depicting their characteristics and inequities, and for exploring ideas about how societies might be understood” claiming that “how communities are imagined” is central to understanding social practices [(41) p. 1852]. Precedent has been set for using fiction from a sociological perspective in a number of recent publications on the sociology of sport (42, 43), and this project travels along a similar trajectory.

The four novels explored here were selected for analysis based on their sustained use of either parkrun, or Couch to 5 K, for plot and character development. All four novels are set in contemporary British contexts. Although other recent novels mention parkrun and/or Couch to 5 K — for instance Elle Spellman's *Running into Trouble* (2020), Jo Clegg's *The Summer Holiday* (2021), and Ross Greenwood's *The Soul Killer* (2020), the four novels selected for analysis here make use of these as substantial tropes throughout. All the novels selected were published during the years of 2019 and 2022 making them recent additions to the literary marketplace and best positioned to engage with topical issues.

Although the textual analysis performed on these novels was focused on issues that emerged from reviewing relevant scholarship on parkrun and Couch to 5 K — namely the themes of health promotion, individual transformation, and community building — the method employed was inherently exploratory in nature. As Belsy suggests, “it is the textual analysis that poses the questions which research sets out to answer” as “the reverse process tends to distort the text.” According to conventional approaches in literary criticism, “the text has priority” and “sets the agenda” [(44) p. 171]. One of the defining features of literary scholarship is that there is no singular “correct” reading of a text (45), but rather various, sometimes conflicting and competing interpretations. As such, it should be recognised that the analysis presented below “is not exhaustive: it does not embrace all the possible readings, past and future” [(44) p. 169].

## Representing parkrun and couch to 5 K in the popular novel

The ensuing analysis of the four key texts explored in this article — Wake's *Saturday Morning Park Run* (2020), Park's *A Run in the Park* (2019), Boleyn's *Coming Home to Cariad Cove* (2022), and James's *I Follow You* (2020) — is arranged thematically around the categories of health promotion, individual transformation, and community building and chart a shift from individual responsibilisation to the social underpinnings of running initiatives. One of the key findings of this analysis is that fictional texts representing the 5 K journey frequently mimic instructional manuals of kinds, in which the nuts and bolts of Couch to 5 K and parkrun, respectively, are presented to the reader. As such, these fictional quasi-instructional manuals then serve to impart collective tacit knowledge to an implied running community. I argue then that not only do these texts frequently operate as health promotion tools in themselves, but they can help familiarise would-be runners with how parkrun and couch to 5 K work and equip them with some of the knowledge that they might need to confidently begin their own running journey.

## Health promotion

Previous scholarship on the mass-participation 5 K run frequently underscores improving physical and mental health as a driving force for individuals to begin running (25, 26), at the same time as highlighting dominant rhetoric in public health initiatives which tend to focus on individual responsibility for health at the expense of social aspects (27). It is not surprising then that fictional manifestations of both parkrun and couch to 5 K habitually draw on such tropes. *Coming Home to Cariad Cove* is a case in point. In the acknowledgements to her novel*,* Boleyn extends a thanks to the NHS, not least in the creation of the Couch to 5 K app which initiated her into the world of running [(46) p. 318]. Much like the implied author here, Boleyn's protagonist Ffion uses running, and the couch to 5 K programme, as part of her recovery after the death of her husband and her return to her parents’ house in Wales. Through the use of a Couch to 5 K app Ffion, along with her sister Mari, who is also going through a transitory period in her life as she emerges from postnatal depression and grapples with marital problems, make the journey from unhappy and solitary non-runners, to more balanced self-identified members of a running community. Couch to 5 K features early in the novel with Joe, the PE teacher love interest of Ffion, extoling the virtues of the programme, which is according to his aunt, “all the rage” [(46) p. 19]. Joe, an experienced runner, mentions to his aunt that he encourages both staff and students at his school to follow the programme if they want to improve their fitness (46)) p. 19]. Replicating dominant ideologies it is, according to Joe, a perfect way to begin running “at any age” [(46) p. 20]. The novel reiterates the kind of rhetoric of inclusivity and accessibility that is frequently remarked upon in 5 K scholarship (1, 16, 21), with Joe being the mouthpiece for this message.

The novels explored here all begin in some way with individual struggles. For instance, in *Coming Home to Cariad Cove,* initially Ffion and Mari are prompted to begin the programme to improve their physical health when they realise that they are not as fit as they would ideally like to be. Mari is spurred on to begin running because she is unhappy with her post-partum body which is “knocking [her] confidence” [(46) p. 88]. Mari declares that “[i]t's not about being skinny, it's about feeling more like me” [(46) p. 88]. Likewise, Ffion too shares a sense of not feeling like herself [(46) p. 89]. If Ffion and Mari have lost touch with their true selves and feel incomplete in some way, Joe, the existing runner, functions as a counterpoint and the epitome of good health and completeness. Joe runs because it helps him to “stay fit and healthy” both in body and mind [(46) p. 86]. As well as the physical benefits, Joe runs “because of the freedom he felt when he ran and became one with movement, with the beating of his heart and his steady breaths, the regular rhythm of his trainers as the hit the ground and pushed him on” [(46) p. 86]; running generates a sense of wholeness and connection with the world around him.

Claire, the first-person protagonist of Wake's 2020 feel-good novel *The Saturday Morning Park Run,* is also shocked by her lack of fitness when she decides to begin running after a mental breakdown. The novel follows Claire and her love interest who is also experiencing mental health problems after the loss of his job and charts their journey to recovery and ultimately love. The plot follows the pair and their journey to set up a new local parkrun. They go from unfit workaholics, to healthy balanced individuals in a novel which resembles what could easily be promotional material produced by parkrun itself. In the process they rope in various lonely individuals, and even a stray dog. Claire, in “[a]ttempting a gentle jog” in the early pages of the novel, finds herself “[b]ent double” and gasping for breath feeling like she is “[d]efinitely dying” [(47) p. 62]. She is disgusted by her own lack of fitness reflecting that she “hadn't even run that far” [(47) p. 62], and feels that she needs to “sort [her]self out” and get “back on track. Proper exercise, proper meals, just like the doctor had ordered” [(47) p. 73]. Claire's uncomplicated encounter embodies the kind of rhetoric dominant in the public health approach that foregrounds individual responsibility for health (19); Claire's individual lifestyle choices in which she has placed excessive value on career success and a status symbol home ultimately led to her neglecting her own physical and thus mental wellbeing. The underlying message of the novel is that, at least in part, Claire is, in the words of Brown, Maslen, & Savulescu (2019), a “morally responsible” agent accountable for her own current situation [(20) p. 114]. Claire largely blames herself for her current state of (un)health and the multifaceted social factors behind this remain in the background.

In *Return to Cariad Cove,* Ffion and Mari settle on running 5 K as a suitable and achievable goal but when they arrive at their first scheduled training session with no particular plan, they are once again confronted with their lack of fitness and disheartened by the negative reinforcement that they get on their first attempt: “I was so excited about trying to get fit” says Mari, who recollects the other women she has seen who “make it look easy”. Ffion is a little more positive: “We’re not rubbish at all. That was just our first attempt” [(46) p. 96]. However, despite her resolve, it turns out that the two women need a little more help than this to achieve their goal. Thankfully, they bump into Joe on the beach where they are running and he introduces them to couch to 5 k as “a great way to start running” [(46) p. 99]. Likewise, it is the 5 K goal that Claire in *The Saturday Morning Park Run* focuses on, and in the short space of time that she manages to build up to running 4 K without stopping Claire finds both her physical and mental health have improved dramatically. Running, she reflects, had given her “a sense of purpose […] and a goal to strive for” [(47) p. 159]. Even though running is portrayed in a positive light in both *Coming Home to Cariad Cove* and *The Saturday Morning Parkrun,* it is initially, prefigured as an individual concern; the characters are individually responsible for their current physical shape, and as such are individually responsible for rectifying this into a socially acceptable mould.

James' 2020 crime novel *I Follow You* offers a counterpoint to the largely positive fictional representations of running that appear predominant. *I Follow You* utilises running as a central trope in this novel which explores how a stalker exploits a social media exercise app to track his victim through the aid of running route and time data. Here the theme of health promotion is present, but in a rather different guise. Georgie, the fitness instructor who is stalked by the murdering gynaecologist Marcus, chose her profession because of her calling to help “people — particularly those at risk from previous sedentary lifestyles — to get fit” [(48) p. 22]. Georgie's meets her clients in an isolated hotel gym which is closed for the winter season, and the physical setting for her one-on-one personal training sessions symbolically mirrors the underlying message of individual health responsibilisation; health and wellbeing is the responsibility of the individual.

Exercise as a solitary and individual endeavour takes on a sinister slant right from the start when Marcus's wife warns him to take it easy because being sedentary, diabetic, and overworked and overweight “he is in the prime early heart-attack band” [(48) p. 31]. Exercise here is framed not as something life-enriching, but something potentially life-threatening. Having chosen Georgie as his victim, Marcus decides to get fit by running using an app called Run Master, but for him, exercise is not a panacea, it only fuels his obsession. Marcus compulsively tracks Georgie, taking note of her personal bests, and parkrun times, with the goal of beating her times, an aspiration which in reality is unachievable for the out-of-shape doctor, even when Georgie becomes pregnant. It is the 5 K distance that Marcus fixates on, though he shows how out of touch with the running scene he is by still thinking in imperial measures as he reflects on one of his early runs: “This new running community he had discovered referred to distances in kilometres — and 5 k was the equivalent of 3.1 miles” [(48) p. 57]. Despite the sinister tone in *I Follow You,* the novel still partially serves an instructional function, disseminating a kind of collective tacit knowledge, for would-be runners on how both parkrun and social media tracking apps work, even if this comes with an underlying message of “readers beware.” Other texts explored here, for instance *Coming Home to Carid Cove,* have a less complicated quasi-instructional tone*.* For instance, after some research into the programme, Ffion and Mari make a renewed attempt to start running, and Boleyn's novel offers a detailed account of how they make use of the Couch to 5 K app to facilitate their journey. For readers who might be unfamiliar with how the programme works, the novel clearly describes all of the steps in day one of the plan. Ffion and Mari provide commentary on the method which serves as explanation both to each other within the text, and for the reader [(46) p. 120]. As Bairner and May proclaim, fictional narratives can function as indicators of “how communities are imagined” and thus pave the way to gaining a more profound understanding of the operation of social practices [(41) p. 1852]. The pedagogic quality infused to varying degrees in the novels explored here imagines a community eager to share the rules, both tacit and explicit, of a particular community of practice. Furthermore, in addition to imagining such a community, these texts could actually have a role in the creation of such “real world” communities since they convey certain sets of values and behaviour which constitute social practice.

## Individual transformation

The theme of individual transformation features commonly in previous scholarship on the 5 K run, with McKendrick, Bowness, and Tulle arguing that events such as parkrun can function as catalysts in an individual's transformation from non-runner to runner (21). Furthermore, as Sharman, Nash, & Cleland explore, it is not just the transformation into simply a runner that is important, running initiatives can also spark a transformational journey from solo runner into membership of a wider running community of practice (33). Here I find that transformation on both these levels forms another common trope in 5 K fiction. Although it is frequently the case that the characters in novels hold themselves accountable for their own state of health at the beginning of the narratives, as they transform into runners they tend to envisage their identities as part of a collective. In this sense, the individuals' transformations can be roughly equated with a move from individual health responsibilisation to social practice (27). An object lesson of this can be found in David Park's collection of short stories. *A Run in the Park* is a more literary, and multi-layered text than the three others explored here, but similarly, it is built on a 5 K journey, this time a group of disparate individuals completing the Couch to 5 K challenge together. The ten stories were originally commissioned by BBC Radio 4 where they aired over a ten-week period, roughly mirroring the time-span of the couch to 5 K programme itself. In printed form, the collection reads more like a novella in which the narrative is constructed out of multiple first-person accounts from the participants of the Couch to 5 K challenge; there is a retired depressed widower who lives off fast food, a middle-aged woman estranged from her adult daughter, an engaged couple having doubts about their upcoming nuptials, and a Syrian refugee. The protagonist is Maurice, the retired widower with whom the novel opens.

The opening line of *A Run in the Park* cements the importance of the theme of identity and individual transformation by affirming Maurice's initial non-runner identity: “I’m a big Bruce Springsteen fan — have seen him live twice — but no matter how I look at it, there isn't any way I was born to run” [(49) p. 1]. For Maurice then, “this unexpected impulse to take up running and try this Couch to 5 K thing goes against the grain of who [he is]” [(49) p. 1] Maurice's urge to begin running is driven by his desire to escape his current state of melancholy. Maurice perceives running to be a way in which he can “kick-start [himself] into forward motion” to be able to “make it through each coming day” [(49) p. 2]. For Yana too, the Syrian refugee who narrates several of the stories in the collection, running is a means of escape, as a way to flee from crisis in a very literal sense: “in times of troubles such as ours, being able to run is no bad thing” [(49) p. 31]. Yana feels at her “safest and most happy” when she is running and senses in each stride a sense of hope [(49) p. 32]. Yana's motivation to run as a means to reach a better and safer place for herself and her family embodies a unique variety of moral responsibilisation. Yana feels in herself a moral responsibility to be physically capable of escape from danger, not only for herself but as a means to protect her family and, for her, the ability to run is equated with survival. Yana, in contrast to Maurice, has running already built into her identity — she is in essence a lone runner despite partaking in a group running programme — but for Maurice, he needs the support of the group to cultivate his running identity.

A similar key theme of individual transformation enabled by a supportive group environment is also present in *Coming Home to Cariad Cove.* As Ffion advances through the couch to 5 K programme she grows progressively stronger both physically and mentally. By the mid-way point in the novel Ffion is transforming into a runner. By week five of the nine-week programme “her body was changing, adapting” [(46) p. 157]. As she prepares for eight minutes straight of running as part of week five's agenda she experiences “a jolt of anticipation then a flutter of nerves” as she sets off determined to succeed [(46) p. 157]. It is on this run that she first experiences the exhilaration of running: “As the endorphins kicked in, a sense of euphoria flooded through her and goosebumps rose on her skin. *This was it!*” [(46) pp. 158–9]. It is this run that precipitates her development in terms of becoming a runner: “This was why she would keep running” (46)) p. 159]. One indicator that Ffion in particular has come to identify as a runner is the way in which she perceives herself as part of a broader running community. The more Ffion runs, the more she becomes aware of other runners: “It had seemed there were more people out running too, or perhaps she just noticed it more now, kind of like when you bought a car, you’d see more of that model around” [(46) p. 281]. As a runner, she is part of an unofficial club of sorts. It is when she finally manages to run a complete 5 K with ease that her running identity is now cemented: “Ffion was alive and she was a runner” [(46) p. 281]. Ffion evolves through the course of the novel from an individual to be held accountable for her own physical state, to a member of a running community of practice.

Claire's path to becoming a “runner” and being initiated into a community of practice in *The Saturday Morning Park Run* is not dissimilar to that of Ffion's. Even though, by the novel's mid-point, Claire is regularly running in her local park she does not categorise herself as a “proper runner”, and it is only when she takes part in a parkrun that she begins to feel a sense of belonging that serves as a catalyst for her own individual transformation making her feel as if she “belonged.” This sense of belonging is spurred on by the fact that she notices that “there were plenty of people who didn't look anything like ‘proper runners’” [(47) p. 201]; in other words, Claire has acquired the tacit knowledge that runners do not have to look like stereotypical runners and consequently with this updated notion of what a runner looks like, Claire has given herself permission to enter the club.

For the implied author of *The Saturday Morning Parkrun,* parkrun has apparently too been transformational. Wake is unashamedly a parkrun enthusiast as she announces in the acknowledgements to her novel: “You might have guessed I’m a bit of a Parkrun fan, although I definitely fall in to the category of plodder rather than runner” (47). As well as identifying as a parkrunner (or parkplodder) herself, Wake extolls the virtues of the organisation. The parkrun organisation, writes Wake, “has done a wonderful job encouraging more people to improve their health and well being by taking up running” (47). Furthermore, in a blog entry dated 16 January, 2021, Wake writes of her astonishment at “having the no.1 best seller in the Amazon category of Running and Jogging! Not something I imagined would ever happen when I first started doing the Park Run, with my husband, in our home town of Tring, just over two years ago.” For Wake then, parkrun has not only transformed her into a parkrunner but a running writer, cementing her belonging in the running community. This input from the implied author serves to highlight the possible role that both peritextual and extratextual dimensions can bring to an understanding of a fictional text as it becomes an integral part of the meaning generated (50).

## Community building

Intricately connected to well-being, and to the theme of transformation, are the opportunities that both parkrun and Couch to 5 K offer for community engagement, and this is a common theme in previous scholarship (25, 26). In *Coming Home to Cariad Cove* running is not only represented as a means for individual transformation, but a way to engage in the community, and the process here is somewhat cyclic in nature. One of the central messages in the novel is the value of helping others: “helping others was meant to be good for you” [(46) p. 46]. In order to raise money for an animal shelter Joe and Ffion organise a community run. Reading with the grain, the novel shares the ethos that running is for everybody, and inclusivity and accessibility are key concerns in the event's planning stages [(46) p. 188]. Later in the novel Joe and Ffion are interviewed for a local newspaper about their impending community run during which they show their gratitude to the help that they have received from local people and businesses. The response from the community was, in Joe's words “overwhelming” [(46) p. 278].

It is Hilda, the eccentric elderly sidekick, in *The Saturday Morning Park Run* who serves as a pivotal figure in terms of community building and it is she who initiates setting up a new local parkrun. Here she acts as a kind of parkrun ambassador, explaining the concept and pontificating on its virtues [(47) p. 125]. Much like Joe in *Coming Home to Cariad Cove,* Hilda, the pensioner who runs daily, spouts a popular kind of public health rhetoric about running: “Everyone knows more people need to exercise. Running can help prevent cardiovascular problems and strokes” [(47) p. 126]. In emphasising the health benefits for “everyone”, *The Saturday Morning Park Run* moves beyond the individual towards the collective. After delving further into the operation of parkrun events, Claire takes the ambassador role from Hilda providing further explication on what setting up a parkrun involves. Claire is “staggered by just how big and successful the whole thing was. […] There were over one thousand events in twenty-two countries and six million registered runners” (47)) pp. 152–3]. When the idea is proposed to the local council, they too are supportive of the idea with a representative who Claire approaches stating that it would help address various council initiatives such as encouraging exercise as well as bringing in more visitors to a currently underutilised local park [(47) p. 163].

It is when Claire travels to take part in her first parkrun by way of preparing for setting up a local event, that she truly comprehends the sense of community that parkrun can embody. She is initially surprised by the fact that it is not just seasoned runners at the event, but instead “a real mix of shapes and sizes and ages” [(47) p. 189]. During the run, the sense of “camaraderie” strikes Claire [(47) p. 193]. However, “the moment that [Claire] fell in love with the parkrun” was when a fellow runner whom Claire is doing her best to catch up with slows her own pace to cross with Claire over the finish line saying “We go over together” [(47) p. 198]. This “in it together” attitude permeates the novel and is associated not only with those who run, but with those behind the scenes who make parkrun possible. For instance, it is the process of planning parkrun that brings together a “disparate group” who are all “working towards a common cause” prompting Claire to reflect “*I belong. These are my people*” [(47) p. 341].

*A Run in the Park* takes a somewhat different approach to exploring the benefits of community. Maurice's melancholy permeates his sections of narrative in the collection and he takes an initially cynical attitude to the “in it together” (25) ethos of the running group but at the same time surrenders himself to its force. For instance, during the first training session Maurice states that he likes the group leader “because she says all the right things, tells us that we’re all going to help each other, that we can do it” [(49) p. 8]. For Cathy who narrates the second story in the collection, it is the *we,* the being part of a group, which is fundamental to her running journey. Cathy describes how, on the second run of the programme, the group each clad in “high-viz tops” form “a luminous little phalanx running the city's streets” [(49) p. 18]; for Cathy, as is the case for the other members of the group, there is safety in numbers. Being part of the couch to 5 K group enables Cathy to begin thinking of herself as a runner too. Cathy reflects on how being acknowledged by other “proper” runners that they encounter, grants them “admittance to their exclusive club” [(49) p. 8]. As the group near the end of their couch to 5 K journey and the looming parkrun that will mark their completion approaches, the group pull together to an even greater degree. Cathy remarks that on the day of the parkrun “when we complete the run it isn't over, because we’ll wait at the finish line and encourage home every single one of our group” [(49) p. 83]. It is Pauline, the leader who is the glue that holds the group together and it is from her that the rhetoric of being “in it together” emanates [(49) p. 87] *A Run in the Park* is self-conscious in its sometimes cynical representation of this kind of rhetoric with Cathy ruminating on the idea that perhaps “Pauline would be the best prime minister we could ever have, telling this nation what it needs to hear. The things that would make it better at this time when everyone seems to be pulling in different directions” [(49) p. 88]. Though there is a somewhat rebellious part of both Cathy and Maurice who scoff at times at the delivery of the public health message, there is a bigger part of both that recognises that this is ultimately good for them, and for society at large.

The collection concludes with Maurice who does not manage to make it to the final 5 k run because that evening he finds himself rescuing his daughter and her children who are fleeing from an abusive relationship. On the last page, Maurice gets a knock on the door, and he opens it, still kitted out in his running gear, and finds it is the rest of the couch to 5 K group who have come to make sure he gets his run:

As I put on the head torch, I see that they are all there and I thank them. Then we slip through a side gate into the sleeping park and Pauline asks me if I'm ready. They circle round me, hands on my shoulders like I'm a boxer going into the ring, and then we set off, a bright cohort of light moving through the darkness, in a world that is slowly drawing in. And I feel sure somehow Mina is watching, watching and smiling as I do this dancing in the dark for her, while we run on, banishing the shadows, our lights showing us the way” [(49) p. 99].

By the end of *A Run in the Park,* Maurice drops his cynicism and yields to the power of the group which acts as a kind of armour against the world. In these closing lines, Maurice is subsumed by the group and the use of figurative language which imagines the running group as a “bright cohort of light” moving in unison completes the transformation from the individual to the collective.

In *I Follow You,* however, this process of transcending the individual and becoming part of a collective never happens. For the murderous doctor Marcus, community is seen as a threat, and as such Marcus never allows himself to transcend the boundaries of the individual. In the novel parkrun serves as the backdrop for a pivotal chapter. In the preceding chapter, Marcus learns through a social media post that Georgie will be taking part in the event the following morning [(48) p. 128]. Marcus shows up to the event which ominously takes place “in the driving rain and ferocious wind” [(48) p. 129]. The parkrun starting line is realistically presented as a clamour of activity [(48) p. 129]. However, this relatively neutral depiction soon becomes menacing as Marcus “bullie[s] his way through the hundreds of people” in search of Georgie. In a far cry from the parkrun ethos of inclusion and friendliness that is echoed in the feel-good novels I explore above, Marcus finds his ego taking a beating as he collides with another runner and takes a fall. Marcus heaves himself up as others “were pounding past him on either side” and continues “trudging up the hill, head bowed against the stinging, blinding rain” [(48) p. 133]. The other participants are, to Marcus, described in unsettling terms; Marcus is towards the back of the pack of runners, and envisages those who are behind him as a sort of enemy gaining on him. In particular, the depiction for a “woman with a pushchair and gaining on him fast” appears especially sinister, with the baby seated in pushchair “protected by a crinkly see-through rain cover, look[ing] like a ready meal from the chill section of the supermarket, [Marcus] thought. *Microwave five minutes*” [(48) p. 133]. The whole experience, through Marcus's perspective, is hellish, with his fellow participants not comrades, but rivals as he is unable to assimilate his identity with the collective.

In the following chapter when Marcus receives his automated message from parkrun detailing his time — “Hello Marcus. Jersey parkrun results for event #174. Your time was 00:38:20” — he is taken aback, “angry and ashamed of his performance in equal measure” [(48) p. 138]. Marcus vows that he will improve his performance to catch Georgie. Marcus, however, fails to match Georgie on a level playing field and takes desperate and evil measures to try and make her his when he purposefully neglects to treat a fatal injury when operating on Georgie's fiancé after a freak plane accident, and then murders a medical intern who threatens to expose his misdeeds, as well as mixing up biopsy samples leading the pregnant Georgie to assume she has cervical cancer. With her fiancé in the intensive care unit fighting for his life, and after the discovery of the body of Marcus's murder victim, one of the ways her despondency is made manifest is in her failure to make it to parkrun [(48) p. 315]. Ultimately, Marcus is discovered, and the novel has a happy ending for Georgie, her fiancé, and their baby with the story coming full circle back to Georgie's remembrance of the event that sparked the entire series of events, Marcus nearly mowing her down in his Porsche as she is out running. Although for Marcus, the villain of the story, the running community is threatening as other people in general are his enemy; this does not imply that the overarching message of the novel is negative towards running. The underlying message of the narrative might very well be instead pro-community. Marcus, who we are not invited to empathise with in the novel, embodies the dangers of going alone; he is both a threat to others as much as he sees others as a threat to himself.

## Discussion and conclusion

The goal of this project was to explore how fiction centred on parkrun and Couch to 5 K imagines running communities and how it reflects, and thus potentially transmits, certain sets of assumptions and values embedded in real-world communities of practice. The method for analysis was tentative in nature based on the assumption that it is the questions that emerge from textual criticism, rather than concrete answers and recognises the subjective and situated nature of the given interpretations. The above literary analysis has demonstrated how key themes of health promotion, individual transformation, and community building are variously manifested in fictional work. What was uncovered during the process of analysis was that oftentimes, for instance in *The Saturday Morning Park Run* and *Coming Home to Cariad Cove,* these manifestations unequivocally reinforce positive messages about the mental and physical health benefits of running and engaging in the running community, and offer an uncomplicated depiction of the transformation from non-runner to runner. What is worthy of further consideration is how these imagined communities can contribute to understanding social practice (41). In the case of the two feel-good novels explored here, the running communities are represented as rather one-dimensional as necessitated by the genre. Although they fail to offer a realistic rendering of the complexities of lived social realities, they perhaps instead function to illustrate an idealised world, one in which the perceived positive values of a community are condensed into a bite-sized and more readily digestible medium.

However, in other texts, this process is more complex. For instance, in Park's collection, although the protagonist finds comfort and protection in his running group, relinquishing his cynicism is not easy for him. Although the collection ends on a positive note, it maintains a sense of realism in that it does not represent running as a panacea, but rather a coping mechanism in a world of continued challenges. At the far end of the scale from the wholly positive representation is the deeply cynical *I Follow You.* Much of the difference between the texts explored here can, at least in part, be attributed to genre; I suggest that feel good novels, as the genre label would suggest, are much more likely to present an uncomplicated positive and life-affirming message, whereas more literary texts can be expected to approach themes with more nuance and crime fiction more inclined towards a darker representation. In their own ways though, each of these genres can deliver variations of a positive (and dominant ideology affirming) message to their target audience in a way that respects genre conventions.

Despite the degree of complexity with which the message is delivered in the four novels explored here, one key point to emerge is that of the individual vs. the community in terms of responsibility for health. As the above analysis has demonstrated, Claire and Ffion, the respective protagonists of *The Saturday Morning Parkrun* and *Coming Home to Cariad Cove,* undergo a transformation from a solitary existence characterised by both mental and physical health problems, to members of a community where responsibility for wellbeing is a shared concern. They find solace in belonging to a group where the “in it together” (25, 27) mentality bolsters their view of health as something that belongs to communities, not just individuals. Both Claire and Ffion are supported by a running community and what were initially individual goals become communal ones. A similar journey is undertaken by Maurice in *A Run in the Park,* which despite its more realistic depiction of a messier and more complex social reality, still arrives at a similar lesson; he only achieves his individual goal through the support of his running group as they ultimately become one at the novel's conclusion. The figurative language which typifies the more literary tone of *A Run in the Park* allows for a more nuanced and sensory of delivery of this message; — at the end of the novel the reader sees not a group runners but a bright ball of light, and feels the protective force of this. In *The Saturday Morning Parkrun* and *Coming Home to Cariad Cove,* the reader is *told* what the message is, in *A Run in the Park,* this is instead intuited.

As Herman and Vervaeck assert, “[r]eproduction and contestation of ideology are at the heart of literature” (51). The four novels explored here initially seem to replicate, to an extent, dominant ideologies present in mainstream health promotion rhetoric, particularly those values related to health responsibilisation. In spite of this, the novels also typically function to contest values which centre the moral imperative of the individual; the underlying message reproduced in all four texts represents a more forward-thinking approach to health promotion which emphases well-being as a community project (27). There is little in all four texts, despite their genre differences, which challenge this more progressive ideological perspective. However, the question of how these ideologies are refracted back into the social world is beyond the scope of this present investigation. Reading, like running, is an inherently social activity and meaning making is something that happens collectively amongst ideologically situated readers.

The aforementioned goal of this project was to look at how imagined communities operate in novels, but what this analysis can only guess at based on the method employed is how such narratives might fit into the multifaceted generation of collective tacit knowledge within particular communities of practice. A logical next step is to investigate where then the reader fits in this discussion. In this present paper which has predominately been a literary analysis demonstrating how key themes present in scholarship on parkrun and Couch to 5 K have been variously transformed into tropes in fictional works, potential for engaging the reader in conversations about wellbeing has been recognised. Seeing that previous research has suggested that fictional texts are more likely to facilitate personal growth and be a catalyst for change than non-fictional texts (52), a natural progression of this work would be to explore how readers, both non-, new, and experienced 5Kers, interact and engage with fictional texts.

## Data Availability

The original contributions presented in the study are included in the article/Supplementary Material, further inquiries can be directed to the corresponding author/s.

## Footnotes

^1^For instance, K.C. Hill's *Run Your Ass Off: Couch to 5k - The No-Nonsense Beginner's Guide to Running* (2020). Debra Bourne's *parkrun: much more than just a run in the park* (2014) charts the rise of the parkrun movement and has been joined by more recent works such as Alison King's *A-Z of parkrun Tourism UK & Ireland* (2021) and Aileen Jones's *how parkrun changed our lives* (2020).
